# Nasal Cavity Dimensions and Pharyngeal Airway Space in Simple Snoring: A Cross-Sectional CT-Based Study

**DOI:** 10.3390/medicina62091798

**Published:** 2026-09-18

**Authors:** Jeong-Whun Kim, Sung-Woo Cho, Hyun Jung Kim

**Affiliations:** 1 Department of Otorhinolaryngology-Head and Neck Surgery, Seoul National University Bundang Hospital, Seoul National University College of Medicine, Seongnam 13620, Republic of Korea; kimemails7@gmail.com (J.-W.K.); 66163@snubh.org (S.-W.C.); 2 Department of Otorhinolaryngology-Head and Neck Surgery, Gyeongsang National University Changwon Hospital, Changwon 51472, Republic of Korea

**Keywords:** nasal cavity, sleep-disordered breathing, snoring, pharyngeal airway space, minimum cross-sectional area

## Abstract

*Background and Objectives:* The anatomical relationship between nasal cavity dimensions and the pharyngeal airway space (PAS) in simple snoring remains poorly defined. We investigated the association between nasal cavity dimensions and the PAS in adults with simple snoring using computed tomography (CT), focusing on anatomical correlations. *Materials and Methods*: This cross-sectional study included 83 adults with simple snoring and nasal obstruction and a confirmed apnea–hypopnea index (AHI) < 2, enrolled between 2007 and 2022. Polysomnography and CT were performed to measure nasal and pharyngeal airway dimensions. The minimum cross-sectional areas (mCSAs) of three nasal cavity segments and pharyngeal airway dimensions were measured on awake CT images. Multivariable linear regression, adjusted for age, sex, and BMI, was used to evaluate the association between the nasal and pharyngeal airway dimensions. *Results:* A larger bilateral narrowest nasal cross-sectional area was significantly associated with a larger PAS mCSA in the full cohort (standardized β = 0.26, *p* = 0.012), after adjustment for age, sex, and BMI. A 10-mm^2^ larger nasal area corresponded to a 3.6% larger PAS mCSA (95% CI, 0.8–6.5%), and the model explained a modest share of the variance (adjusted R^2^ = 0.17). The association did not differ by sex (interaction *p* = 0.25), indicating no evidence of effect modification. *Conclusions:* In individuals with simple snoring, smaller nasal cavity dimensions were associated with a narrower PAS on static CT imaging, although this association was modest and explained only a small fraction of the variation in pharyngeal calibre. These findings reflect anatomical associations and should not be interpreted as evidence of functional airway collapse or disease progression.

## 1. Introduction

Simple snoring is often considered a mild phenotype within the spectrum of sleep-disordered breathing (SDB) [1]. However, the anatomical and physiological characteristics distinguishing simple snoring from more advanced forms of SDB remain incompletely understood. Previous research has largely focused on the pharyngeal airway space (PAS), with CT-based studies revealing significant correlations between reduced pharyngeal cross-sectional area and both sleep-disordered breathing severity and nocturnal oxygen desaturation in obstructive sleep apnea (OSA) [2]. Similarly, reduced posterior airway space and increased hyoid-to-mandibular plane distances have been reported in patients with OSA compared with controls [3,4].

Conversely, studies on the role of the nasal cavity in SDB have primarily examined outcomes following surgical interventions, such as septal deviation correction [5]. Surgical correction of nasal obstruction improves continuous positive airway pressure (CPAP) adherence and subjective sleep quality [6,7,8,9,10]; however, its direct impact on pharyngeal airway dimensions is controversial [11]. Furthermore, in advanced OSA, nasal obstruction appears to play a diminished role in airway compromise, shifting attention to pharyngeal collapsibility [12,13].

Although nasal structural narrowing has been hypothesized to contribute to airway characteristics in milder forms of SDB, such as simple snoring, the supporting evidence remains limited. This stage, where sleep apnea is uncommon, provides an opportunity to examine the anatomical relationships between nasal obstruction and pharyngeal airway structure. Nasal obstruction may induce mouth breathing and mandibular retrograde movement during sleep, contributing to pharyngeal airway narrowing [14]. Understanding the anatomical associations at this stage may provide contextual insights into the upper airway structure in individuals with simple snoring. However, research on the relationship between narrowing of the nasal cavity space and narrowing of the PAS during simple snoring is limited.

This study aimed to explore the correlation between nasal cavity dimensions and the PAS in individuals with simple snoring using computed tomography (CT) imaging.

## 2. Methods

### 2.1. Patient Selection and Data Collection

**Figure 1:**
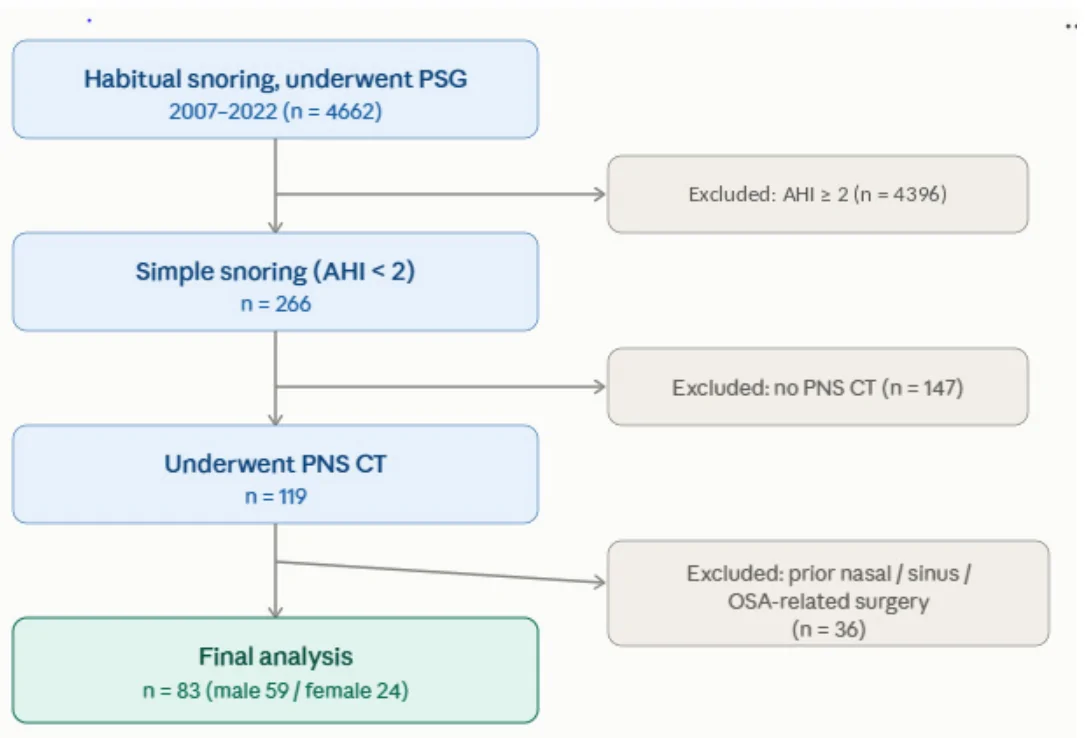
STROBE flow diagram.

This retrospective, cross-sectional study analyzed patients who presented to the outpatient clinic with simple snoring at a single tertiary referral hospital between 2007 and 2022. Patients underwent overnight polysomnography (PSG), and those who reported symptoms of nasal obstruction when routinely questioned about nasal symptoms at the outpatient visit underwent paranasal sinus (PNS) CT to evaluate the nasal cavity. The inclusion criterion was an apnea–hypopnea index (AHI) of less than 2, as determined by PSG. Simple snoring is conventionally defined as an AHI < 5; we deliberately applied the stricter threshold of AHI < 2 to isolate a pure non-apneic snoring phenotype and to exclude the AHI 2–5 range, in which early sleep-disordered-breathing pathophysiology could confound a purely anatomical nasal–pharyngeal association. All consecutive eligible patients over the study period were included, with no further selection. Patients with a history of prior nasal, sinus, or OSA-related surgery were excluded (Figure 1).

Demographic and clinical data were systematically collected for analysis. The study was approved by the Institutional Review Board (IRB No. B-2312-870-102).

### 2.2. Polysomnography and Imaging

All patients underwent Level 1 full-night polysomnography (PSG; Embla N7000, Reykjavik, Iceland) to assess SDB. A single PSG platform and a single hypopnea scoring rule were used consistently throughout the enrolment period. The PSG recordings included electroencephalography, electrooculography, chin and limb electromyography, electrocardiography, nasal pressure transducers, chest and abdominal respiratory inductance plethysmography, and pulse oximetry. PNS CT was performed to evaluate the nasal cavity and to measure nasal and pharyngeal airway dimensions. CT and PSG were performed a median of 5 days apart (mean, 5.3 ± 3.0 days; range, 0–14 days), with CT performed on the same day as, or before, PSG in all patients. CT was acquired using multidetector CT scanners at 120 kVp, with a 2-mm slice thickness and 2-mm interval and a metal artifact reduction algorithm. More than one scanner generation was used over the study period, but the acquisition protocol was standardized and unchanged throughout. The field of view covered the entire craniofacial region, encompassing both the nasal cavity and the pharyngeal airway in all patients. Scans were obtained during quiet breathing, with the patient instructed not to swallow, cough, or move during the scan; the patient was positioned supine with the inferior orbitomeatal line aligned perpendicular to the CT table and no gantry tilt, and with the mouth closed and the mandible in a relaxed, neutral resting position (teeth gently in normal occlusion). The scans were acquired on six CT systems over the enrolment period: Philips Brilliance 64 (Philips Healthcare, Best, The Netherlands), iCT 256 (Philips Healthcare, Best, The Netherlands), Mx8000 IDT 16 (Philips Healthcare, Best, The Netherlands), Iqon Spectral CT (Philips Healthcare, Best, The Netherlands), and Mx8000 (Philips Healthcare, Best, The Netherlands) (82 scans in total), and a Siemens SOMATOM Definition Edge (Siemens Healthineers, Forchheim, Germany) (1 scan). All used a standardized 120-kVp, 2-mm-slice protocol. The distribution of scans across scanner and enrolment era is provided in Supplementary Table S4. Coronal, axial, and sagittal images were analyzed using Picture Archiving and Communication System (PACS) software. All cross-sectional areas and linear distances were measured directly on the PACS workstation in calibrated physical units (mm^2^ and mm) derived from the DICOM pixel-spacing data, at a representative window setting (window width 2500, level approximately 600); because the measured target was the air-filled lumen, which is highly contrasted against the surrounding soft tissue and bone irrespective of the exact window/level, the precise window setting is unlikely to have had a meaningful influence on the measurements. The primary measurements for all patients were performed by an otorhinolaryngologist with 8 years’ experience, using fixed anatomical landmarks and blinded to the patients’ sex and polysomnographic data. For the reliability analysis, a random subsample was additionally re-measured by the same observer and, independently, by a second otorhinolaryngologist. Reliability was assessed in a random subsample of 30 scans (blinded to sex, polysomnographic data, and the original values); agreement was high for all four measurements (intra-observer ICC 0.953 to >0.999; inter-observer ICC 0.953 to >0.999), with ICCs (two-way random effects, absolute agreement, single measurement) and Bland–Altman limits of agreement reported in Supplementary Table S5.

### 2.3. Quantification of Nasal Cavity Dimensions

**Figure 2:**
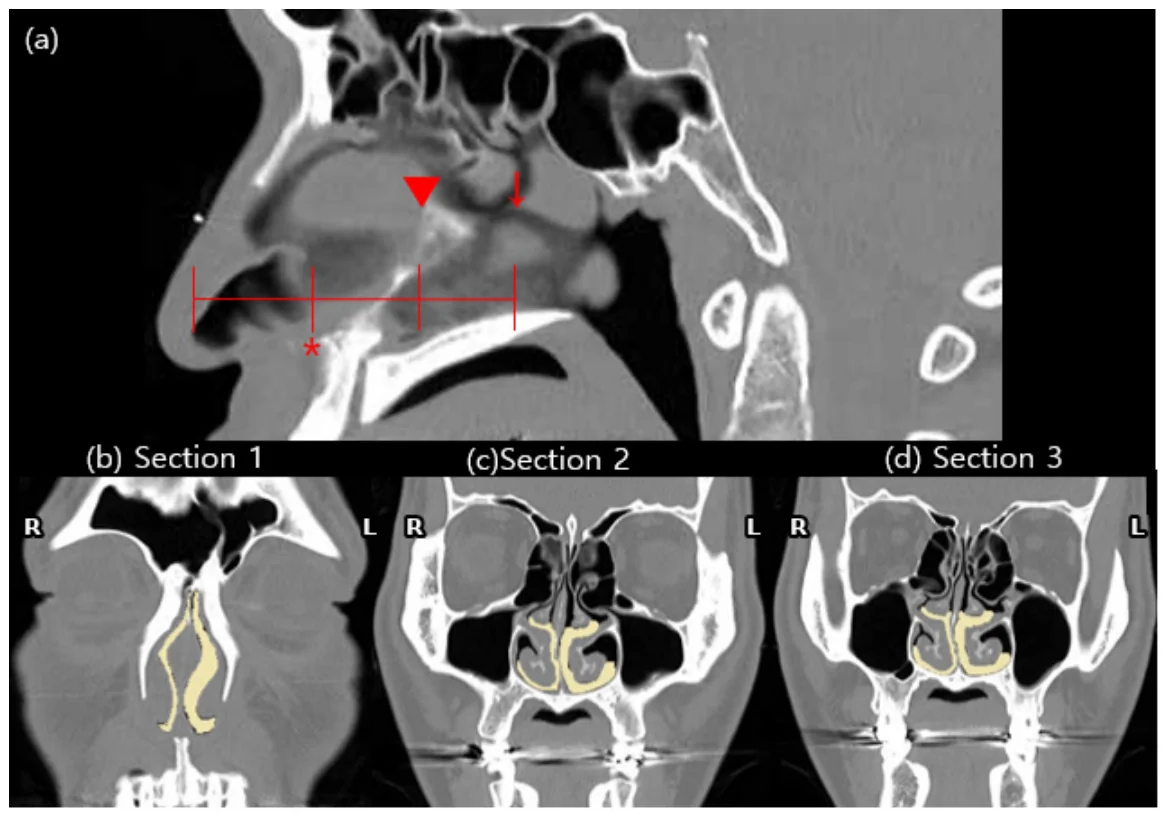
Three distinct sections of the nasal cavity. (**a**) Sagittal CT view (left side) showing the landmarks that delimit the three sections: the anterior nasal spine of the maxilla (asterisk, *), the junction of the nasal septum (arrowhead, ▼), and the anterior end of the superior turbinate (arrow, ↓). Section 1 extends from the nostril to the anterior nasal spine (*); Section 2, from the anterior nasal spine (*) to the nasal septal junction (▼); and Section 3, from the nasal septal junction (▼) to the anterior end of the superior turbinate (↓). (**b**) Minimum cross-sectional area (mCSA) in Section 1 (S1). (**c**) mCSA in Section 2 (S2). (**d**) mCSA in Section 3 (S3). All measurements were performed in calibrated physical units (mm^2^) on the PACS using DICOM pixel-spacing data and are independent of the displayed image magnification. Window width/level ≈ 2500/600.

The nasal cavity was segmented into three anatomically distinct regions. Section 1 (S1), the initial cartilaginous segment, extended from the nostril to the anterior nasal spine and primarily represented the cartilaginous portion of the nasal airway. Section 2 (S2), the bony-cartilaginous segment, spanned from the anterior nasal spine to the junction where the perpendicular plate, vomer, and septal cartilage meet (the nasal septum junction). Section 3 (S3), the bony segment, stretched from the nasal septum junction to the anterior end of the superior turbinate; because the superior turbinate lies within the posterior nasal cavity, S3 includes the anterior and middle portions of the posterior nasal cavity (Figure 2).

The minimum cross-sectional area (mCSA) was measured for each section in the coronal CT views, in both the right and left nasal cavities. For the primary analysis, the bilateral cross-sectional area of the narrowest section was defined as the minimum, across the three sections, of the summed left + right area; this bilateral measure reflects the parallel arrangement of the two nasal cavities and is not distorted by the side-to-side asymmetry produced by septal deviation. The single-side smallest mCSA (the minimum of the six section measurements) was retained for a sensitivity analysis.

### 2.4. Pharyngeal Airway Space Measurements

**Figure 3:**
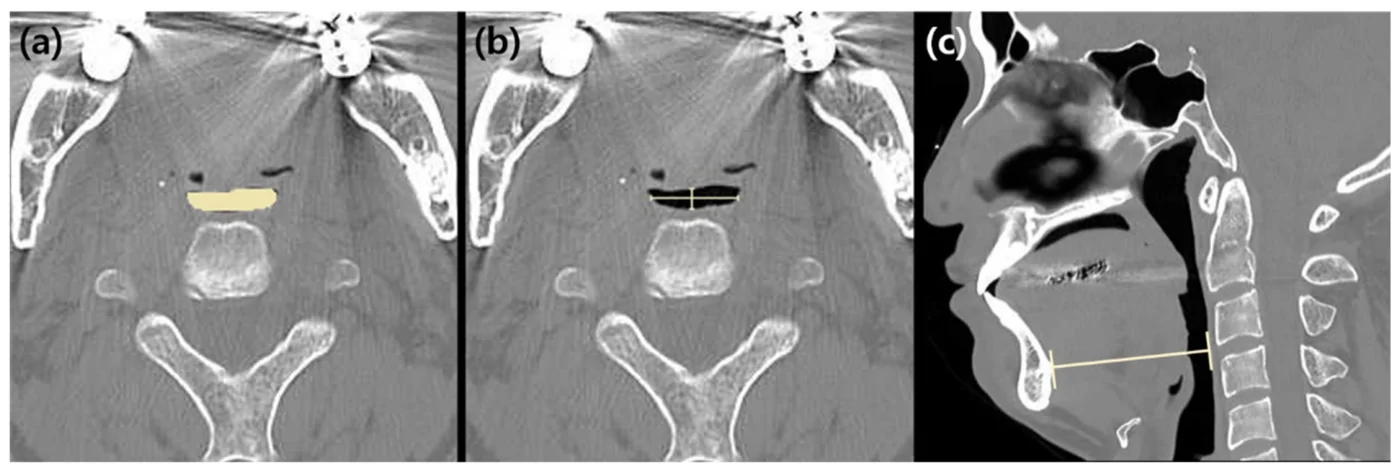
Measurements of the pharyngeal airway space (PAS). Panels (**a**,**b**) show the same axial slice—the retroglossal slice with the smallest cross-sectional area in that patient (bounded by the free margin of the uvula superiorly and the epiglottis inferiorly; approximately the C2–C3 vertebral level, which varied between individuals). (**a**) PAS mCSA: minimum cross-sectional area of the PAS. (**b**) PAS lateral and PAS AP: lateral and anteroposterior diameters measured on the same slice. (**c**) Distance from the genial tubercle to the posterior pharyngeal wall, measured on the midsagittal plane.

PAS dimensions were assessed using four parameters: the minimum cross-sectional area of the PAS (PAS mCSA), the lateral and anteroposterior (AP) diameters of the PAS, and the distance from the genial tubercle to the posterior pharyngeal wall. In each patient, the retroglossal segment—bounded superiorly by the free margin of the uvula and inferiorly by the epiglottis (approximately the C2–C3 vertebral level; the exact level varied between individuals)—was searched for the axial slice with the smallest cross-sectional area, examined at the 2-mm reconstruction interval on the axial plane defined perpendicular to the inferior orbitomeatal line (Section 2.2). The PAS mCSA, lateral, and AP dimensions were all measured on that same slice. The genial tubercle-to-posterior-pharyngeal-wall distance was measured on the midsagittal plane, on which the genial tubercle—a midline bony structure—was clearly visualized (Figure 3).

### 2.5. Statistical Analysis

Continuous variables are summarized as mean (standard deviation) and were compared between sexes using the independent-samples *t*-test or the Mann–Whitney U test according to the normality of distribution, assessed by the Shapiro–Wilk test. To investigate the association between nasal cavity dimensions and the PAS, multivariable linear regression was performed using the enter method, with all covariates entered simultaneously. Because the PAS mCSA was strongly right-skewed (Shapiro–Wilk W = 0.73, *p* < 0.001; skewness = 3.1), the primary outcome was the natural-log-transformed PAS mCSA; the untransformed model was retained as a sensitivity analysis. The primary exposure was the bilateral narrowest-section nasal area, and the model was adjusted for age, sex, and BMI. The bilateral narrowest-section area and the natural-log transformation were adopted during revision in response to peer review and were not pre-specified in the original protocol, in which the single-side smallest area and the untransformed outcome were the primary specifications; these original specifications are retained here as sensitivity analyses. The full set of exposure × outcome-scale specifications is reported in Supplementary Table S2. To assess whether this association could be attributed to body-size scaling, we performed sensitivity analyses in which the primary model was additionally adjusted for height, for neck circumference, for both, and for body surface area (Mosteller formula). To assess whether the primary association was influenced by scanner heterogeneity, we refitted the primary model with scanner entered as a covariate (as scanner model and, separately, as manufacturer) and repeated it within the largest single-scanner subgroup. A sex × nasal-mCSA interaction term was fitted to test for effect modification by sex. In exploratory analyses, the PAS mCSA was regressed separately on the bilateral cross-sectional area of each nasal segment (S1, S2, and S3 in turn), each adjusted for age, sex, and BMI. The association between the bilateral narrowest-section nasal area and PAS mCSA was designated as the single primary analysis. The secondary outcomes (PAS lateral, PAS anteroposterior dimension, and genial-tubercle distance), the segment-specific models, the sensitivity analyses, and the sex-interaction test were exploratory and were not adjusted for multiple comparisons; their *p*-values should be interpreted accordingly. AHI and the snoring episode index were not entered into the regression because the cohort was restricted to AHI < 2, leaving negligible variance in these variables; they are reported only as descriptive characteristics. There were no missing data for any analyzed variable, so all 83 patients were included in every model; no imputation was required. Model fit was summarized by the adjusted R^2^, and multicollinearity by variance inflation factors (VIFs). No a priori sample-size calculation was performed; this was a retrospective analysis that included all consecutive eligible patients over the study period, and the sample size was therefore determined by the available cohort rather than by a formal power calculation. Analyses were performed using IBM SPSS Statistics, version 25 (IBM Corp., Armonk, NY, USA), with significance set at *p* < 0.05.

## 3. Results

### 3.1. Patient Demographics and Characteristics

A total of 83 patients (59 males and 24 females) were included. The overall mean age, BMI, and AHI were 41.2 years, 24.0 kg/m^2^, and 1.0 events/hour, respectively. Males and females differed significantly in BMI (24.3 vs. 23.1 kg/m^2^, *p* = 0.025) and AHI (1.1 vs. 0.7 events/hour, *p* = 0.036), with a borderline difference in age (39.4 vs. 45.7 years, *p* = 0.050). Males were larger on all body-size measures, including height, weight, and neck, waist, and hip circumference (all *p* ≤ 0.012). The snoring episode index, computed as the number of snoring episodes per hour of total sleep time, was 5.5 (5.9)/h overall and did not differ significantly between sexes (Table 1).

### 3.2. Nasal Cavity Dimensions

The mean section mCSAs are shown in Table 2. Sex-specific comparisons revealed no statistically significant differences in the mCSAs across all sections. The single-side smallest mCSA (the narrowest segment across all sections) was 44.4 mm^2^ and did not differ between males and females (*p* = 0.82). The bilateral cross-sectional area of the narrowest section, used as the primary exposure, was 119.2 (52.8) mm^2^ overall and also did not differ significantly between sexes (*p* = 0.54) (Table 2).

### 3.3. Pharyngeal Airway Space Dimensions

PAS measurements revealed significant sex-related differences. The mean PAS mCSA was larger in males (119.4 mm^2^) than in females (73.8 mm^2^) (*p* = 0.020). The AP length of the PAS was greater in males (8.7 mm) than in females (6.9 mm) (*p* = 0.016), and the distance from the genial tubercle to the posterior pharyngeal wall was longer in males (64.0 mm) than in females (55.9 mm) (*p* < 0.001). No significant difference was observed in the lateral dimension (16.7 vs. 15.2 mm, *p* = 0.47) (Table 3).

### 3.4. Association Between Nasal Cavity Dimensions and PAS mCSA

In the multivariable model of the full cohort, a larger bilateral narrowest-section nasal area was significantly associated with a larger (log) PAS mCSA (B = 0.0035 per mm^2^; 95%CI, 0.0008–0.0063; standardized β = 0.26; *p* = 0.012), after adjustment for age, sex, and BMI (n = 83; adjusted R^2^ = 0.17; all VIF ≤ 1.1) (Table 4). In back-transformed terms, a 1-SD larger nasal area (52.8 mm^2^) corresponded to a 20.4% larger PAS mCSA (95% CI, 4.3% to 39.1%; equivalently 3.6% per 10 mm^2^, 95% CI 0.8% to 6.5%); the nasal-cavity term uniquely accounted for approximately 7 percentage points of the variance in log PAS mCSA. Age (β = 0.23, *p* = 0.029) and male sex (β = 0.30, *p* = 0.006) were also positively associated with the PAS mCSA, whereas BMI was not (*p* = 0.12). The association was significant on both the log and untransformed scales (untransformed *p* = 0.001; Supplementary Figure S1). This association was robust to adjustment for body size: adding height, neck circumference, both variables, or body surface area to the primary model left the nasal-area coefficient essentially unchanged (B = 0.0035 in every model; *p* = 0.012–0.013; standardized β = 0.26), and the variance inflation factor for the nasal-area term remained 1.02 throughout (Supplementary Table S3). In the sensitivity analysis using the single-side smallest mCSA on the log scale, the association was in the same direction and remained significant (B = 0.0068; 95% CI, 0.0003 to 0.0134; *p* = 0.041). Across all four combinations of exposure definition and outcome scale, the association was concordant in direction and statistically significant (Supplementary Table S2), although it was weakest for this single-side log combination. Because the primary exposure is the minimum across the three segments, segment-specific models are not independent replications; each individual segment area was nonetheless positively associated with PAS mCSA (S1, *p* = 0.015; S2, *p* = 0.019; S3, *p* = 0.012).

The association was also robust to scanner heterogeneity. With scanner model entered as a covariate, the nasal-area coefficient remained significant (B = 0.0030; *p* = 0.034), and it was essentially unchanged with manufacturer adjustment (B = 0.0035; *p* = 0.014); in the largest single-scanner subgroup (Philips Brilliance 64, n = 43), it was of similar magnitude and direction (B = 0.0030), though not significant at the reduced sample size (Supplementary Table S4).

To assess whether the association differed by sex, a sex × nasal-mCSA interaction term was added to the full-cohort model. The interaction was not significant (*p* = 0.25 for the bilateral exposure; *p* = 0.57 for the single-side measure), indicating no evidence of effect modification by sex. Sex-stratified estimates are therefore reported only as exploratory. For the secondary PAS outcomes against the same bilateral exposure, the PAS lateral (B = 0.033; 95% CI, 0.011–0.054; *p* = 0.004) and PAS AP (B = 0.015; 95% CI, 0.004–0.026; *p* = 0.010) dimensions were significantly associated with the nasal measure, whereas the genial-tubercle distance was not (*p* = 0.42).

## 4. Discussion

Previous studies have indicated the limited efficacy of isolated nasal surgery in managing advanced stages of SDB [6]. Our study shifted focus to individuals with simple snoring, offering insights into the anatomical characteristics observed within the milder end of the SDB spectrum. By investigating the relationship between nasal cavity dimensions and the PAS, this study highlights the potential relevance of nasal anatomy to downstream airway structure.

Nasal cavity and pharyngeal airway dimensions have also been jointly assessed in the orthodontic literature using CBCT, where they have been related to craniofacial morphology and, in some adult cohorts, positively correlated with one another [15]. Those studies were conducted in orthodontic or orthognathic populations selected by skeletal or malocclusion class and analyzed airway volumes in relation to craniofacial structure. The present study differs in both population and question: it examines a defined simple-snoring phenotype (AHI < 2) and directly tests the relationship between the nasal cavity and the minimum retroglossal pharyngeal cross-sectional area—the functionally relevant narrowest point—while adjusting for age, sex, and BMI. It thus extends the observation of a nasal–pharyngeal anatomical relationship beyond craniofacial-morphology-driven analyses into the simple-snoring phenotype.

Unlike previous CT-based assessment of the upper airway in OSA [16], we measured the nasal cavity and the PAS mCSA on the same scans, allowing the nasal–pharyngeal relationship to be assessed directly within individuals. To enhance precision, the nasal cavity was segmented into three anatomically distinct regions: the initial cartilaginous segment (S1), the bony-cartilaginous segment (S2), and the bony segment (S3). This segmentation method, designed for ease of application, ensures anatomical consistency and methodological transparency. Common causes of nasal obstruction—such as septal deviation, septal body prominence, and inferior turbinate hypertrophy [17,18,19]—are captured within the segmental mCSA as luminal narrowing; these features were not separately graded or entered as covariates, which we note as a limitation. Because septal deviation produces side-to-side asymmetry, the primary analysis used a bilateral (summed left + right) measure of the narrowest section, which compensates for that asymmetry and reflects total nasal patency rather than deviation-dependent narrowing of one side.

Overall, the present study demonstrated a statistically significant association between nasal cavity dimensions and the PAS in individuals with simple snoring based on static CT imaging: a narrower nasal cavity was associated with a smaller pharyngeal airway area. These findings reflect anatomical correlations rather than functional measurements of airway behavior during sleep. One possible explanation is that reduced nasal airway dimensions may increase airflow resistance, which has been hypothesized to influence the breathing route and mandibular posture during sleep [14,20]. The activation of nasal receptors during breathing has been implicated in maintaining ventilation and oropharyngeal muscle tone [21], and reduced receptor activation has been proposed to decrease genioglossal activity; however, such mechanisms cannot be confirmed by static, awake CT imaging and remain speculative.

Nasal obstruction has been suggested to promote mouth breathing, which may influence upper airway conditions during sleep. Mouth breathing has been associated with altered upper airway humidification and environment [22], and a previous CT-based study reported a reduction in the upper airway cross-sectional area, particularly in the retroglossal region, when the mouth was opened compared with the mouth-closed condition within the same subjects [23]; and a study that assessed breathing route by capnography during propofol-based drug-induced sleep endoscopy reported that mouth breathing was associated with worse oxygen desaturation and more severe upper airway collapse [20]. Nasal breathing confers a mechanical advantage over oral breathing during sleep, with significantly lower upper airway resistance and fewer obstructive events [24]. However, such mechanisms cannot be confirmed by static, awake CT imaging and remain speculative.

We observed sex differences in the pharyngeal airway dimensions themselves, with males showing a larger PAS mCSA, AP length, and genial-tubercle distance. Sex differences in upper airway anatomy and its relationship to SDB severity have likewise been reported by Mohsenin [25,26], although those studies assessed patients with established OSA (AHI/RDI ≥ 5) using acoustic reflectance rather than CT, in older and more obese cohorts than the present simple-snoring sample. However, a formal sex × nasal-mCSA interaction test was not significant, so we do not interpret the nasal–pharyngeal association itself as sex-dependent; the full-cohort association is the primary finding, and sex-stratified estimates are exploratory only, particularly given the small, underpowered female subgroup (n = 24).

The snoring episode index was included only as a descriptive polysomnographic parameter to characterize the simple-snoring cohort [27]; because the cohort was restricted to AHI < 2, it was not modeled as a predictor. Its lower value here than in the original defining cohort is expected, as that cohort spanned the full OSA severity range whereas the present cohort comprised simple snorers.

This study had several limitations. First, its cross-sectional design precluded causal inference. Second, the cohort represented a selected group of simple snorers with nasal obstruction who underwent CT, which limits generalizability. Because CT was performed only in those reporting nasal obstruction, the analytic sample was likely skewed toward narrower nasal dimensions; this restriction of the exposure range would be expected to attenuate rather than exaggerate the observed nasal–pharyngeal association. A direct comparison with eligible snorers who did not undergo CT was not available; the conservative AHI < 2 threshold further limits generalizability to the broader AHI < 5 population, and extension to the AHI 2–5 range is a direction for future work. Third, pharyngeal airway behavior during sleep is dynamic and is influenced by neuromuscular tone, body position, and sleep stage. Awake CT imaging captures the static anatomy only and should not be interpreted as a surrogate for airway collapsibility or functional obstruction. Although scan positioning was standardized (supine posture, inferior orbitomeatal line perpendicular to the table, neutral mandibular position), cervical flexion and extension were not quantified; because head and neck posture can affect retroglossal airway dimensions, unmeasured variation in cervical posture may have introduced measurement variability in the pharyngeal measures. Fourth, CT was acquired over a long period on more than one scanner generation. Although the acquisition protocol was standardized, we verified that the primary association persisted after adjustment for scanner and within the largest single-scanner subgroup (Supplementary Table S4); the manufacturer was moreover essentially constant (82 of 83 scans on Philips systems), limiting between-vendor differences in reconstruction. We nonetheless acknowledge that residual between-scanner variation cannot be fully excluded. The sample size was not based on a formal power calculation but was fixed by the number of eligible patients; the study may therefore have been underpowered to detect small effects or subgroup differences. In addition, PSG and CT were not performed on the same day (median interval, 5 days; maximum, 14 days); although this short interval makes meaningful anatomical change between assessments unlikely, we cannot fully exclude minor changes in airway dimensions between the two examinations. Finally, the female subgroup was relatively small, potentially limiting sex-specific analyses.

## 5. Conclusions

In individuals with simple snoring, smaller nasal cavity dimensions were associated with a narrower pharyngeal airway space on awake, static CT imaging; this association, although statistically significant, was modest and explained only a small fraction of the variation in pharyngeal airway calibre. These findings describe anatomical correlations and should not be interpreted as evidence of causation, disease progression, functional airway collapse, or clinical predictive utility. Prospective and functional studies are needed to determine whether these anatomical relationships have physiological or clinical relevance in sleep-disordered breathing.

## Figures and Tables

**Table 1:** Demographic and polysomnographic characteristics.

	Overall (*n* = 83)	Males (*n* = 59)	Females (*n* = 24)	*p*-Value
Demographic measures				
Age, mean (SD), y	41.2 (13.4)	39.4 (13.2)	45.7 (13.2)	0.050
Height, mean (SD), cm	168.6 (8.7)	172.9 (5.7)	158.2 (5.1)	<0.001 *
Weight, mean (SD), kg	68.4 (12.6)	72.8 (10.4)	57.6 (11.1)	<0.001 *
Body mass index, mean (SD), kg/m^2^	24.0 (3.5)	24.3 (2.9)	23.1 (4.8)	0.025 *
Neck circumference, mean (SD), cm	35.8 (3.3)	37.4 (2.0)	31.9 (2.4)	<0.001 *
Waist circumference, mean (SD), cm	84.6 (9.4)	87.5 (7.2)	77.7 (10.6)	<0.001 *
Hip circumference, mean (SD), cm	97.0 (7.2)	98.0 (6.1)	94.5 (9.2)	0.012 *
PSG parameters				
Apnea–hypopnea index, mean (SD), /h	1.0 (0.6)	1.1 (0.6)	0.7 (0.5)	0.036 *
Snoring episode index, mean (SD), /h	5.5 (5.9)	6.2 (6.3)	3.9 (4.5)	0.143

SD, standard deviation. * *p*-values indicate significance between males and females. The snoring episode index was computed as the number of snoring episodes per hour of total sleep time.

**Table 2:** Nasal cavity cross-sectional area by sex.

	Overall (*n* = 83)	Males (*n* = 59)	Females (*n* = 24)	*p*-Value
mCSA for S1				
Right, mean (SD), mm^2^	104.8 (45.9)	105.5 (50.0)	103.2 (34.5)	0.806
Left, mean (SD), mm^2^	105.5 (43.9)	108.5 (45.1)	98.3 (40.1)	0.341
mCSA for S2				
Right, mean (SD), mm^2^	75.8 (48.4)	76.2 (50.4)	75.0 (44.2)	0.914
Left, mean (SD), mm^2^	86.5 (50.6)	93.2 (50.5)	70.0 (48.0)	0.058
mCSA for S3				
Right, mean (SD), mm^2^	60.4 (32.1)	60.0 (30.1)	61.6 (28.5)	0.834
Left, mean (SD), mm^2^	63.7 (31.6)	65.7 (30.7)	58.7 (34.0)	0.364
Combined measures				
Single-side smallest mCSA, mean (SD), mm^2^	44.4 (22.7)	44.4 (22.5)	44.4 (23.6)	0.821
Bilateral narrowest-section area, mean (SD), mm^2^	119.2 (52.8)	121.3 (51.5)	114.2 (56.5)	0.543

SD, standard deviation. The bilateral narrowest-section area (summed left + right area of the narrowest section) was the primary exposure; the single-side smallest mCSA was used in a sensitivity analysis.

**Table 3:** Pharyngeal airway space dimensions by sex.

	Overall (*n* = 83)	Males (*n* = 59)	Females (*n* = 24)	*p* Value
PAS mCSA, mean (SD), mm^2^	106.2 (84.8)	119.4 (95.3)	73.8 (35.0)	0.020 *
PAS lateral, mean (SD), mm	16.2 (5.8)	16.7 (6.2)	15.2 (4.7)	0.473
PAS AP, mean (SD), mm	8.1 (2.9)	8.7 (3.1)	6.9 (2.2)	0.016 *
Genial tubercle to posterior pharyngeal wall, mean (SD), mm	61.7 (7.9)	64.0 (7.0)	55.9 (7.1)	<0.001 *

PAS, pharyngeal airway space; PAS mCSA, minimum cross-sectional area of the PAS; SD, standard deviation; AP, anteroposterior. * *p*-values indicate significance between males and females.

**Table 4:** Multivariable linear regression for the association with (log) PAS mCSA (full cohort, n = 83).

Predictor	B	SE	β	95% CI	*p*
Bilateral narrowest-section nasal area	0.0035	0.0014	0.26	0.0008 to 0.0063	0.012
Age	0.0124	0.0056	0.23	0.0013 to 0.0234	0.029
Sex (male)	0.4632	0.1640	0.30	0.1366 to 0.7898	0.006
Body mass index	−0.0325	0.0207	−0.16	−0.0737 to 0.0086	0.119

Outcome: natural-log-transformed PAS mCSA. B, unstandardized coefficient; SE, standard error; β, standardized coefficient; CI, confidence interval. Model n = 83; adjusted R^2^ = 0.17; all variance inflation factors ≤ 1.1.

## Data Availability

The de-identified dataset supporting the findings of this study is available as Supplementary Data. Individual identifiers were removed, and age was banded to minimize re-identification risk.

## Supplementary Materials

The following supporting information can be downloaded at: https://www.mdpi.com/article/10.3390/medicina62091798/s1. Table S1: STROBE Statement—checklist of items that should be included in reports of cross-sectional studies; Figure S1: Regression diagnostics for the PAS mCSA model. Residual-versus-fitted plots, normal Q–Q plots, and residual histograms are shown for the untransformed outcome (panels A–C) and the natural-log-transformed outcome (panels D–F). Log-transformation improved the normality of the residuals (Shapiro–Wilk W: 0.87 → 0.94; skewness: 1.9 → −1.0) and removed the heteroscedasticity present in the untransformed model (Breusch–Pagan P: 0.01 → 0.95), supporting the log scale as the primary specification; Table S2: Association between nasal cavity area and PAS mCSA across all combinations of exposure definition and outcome scale (multivariable linear regression, n = 83); Table S3: Sensitivity of the nasal cavity–PAS association to adjustment for body size (multivariable linear regression, n = 83; outcome = log-transformed PAS mCSA); Table S4: Distribution of CT scans across scanner model and enrolment era (n = 83); Table S5: Intra- and inter-observer reliability of the airway measurements (random subsample of 30 scans). Supplementary Data: De-identified analysis dataset.

## Abbreviations

The following abbreviations are used in this manuscript:

AHI	apnea–hypopnea index
AP	anteroposterior
BMI	body mass index
CPAP	continuous positive airway pressure
CT	computed tomography
ICC	intraclass correlation coefficient
mCSA	minimum cross-sectional area
OSA	obstructive sleep apnea
PACS	Picture Archiving and Communication System
PAS	pharyngeal airway space
PAS mCSA	minimum cross-sectional area of the pharyngeal airway space
PNS	paranasal sinus
PSG	polysomnography
SDB	sleep-disordered breathing
VIF	variance inflation factor
