# Supplementary material for: Nasal Cavity Dimensions and Pharyngeal Airway Space in Simple Snoring: A Cross-Sectional CT-Based Study
Source: Medicina (Kaunas). 2026 Sep 18;62(9):1798. doi: 10.3390/medicina62091798 (PMC13609348; doi:10.3390/medicina62091798)
Supplement: Supplementary file 1 [file medicina-62-01798-s001.zip › 1.Supplementary materials.pdf]

# Supplementary Material

## 1. Supplementary Table S1.

### Table S1. STROBE Statement—checklist of items that should be included in reports of cross-sectional studies

*Manuscript: "Nasal cavity dimensions and pharyngeal airway space in simple snoring: a cross-sectional CT-based study." Page and line numbers refer to the revised manuscript.*

| Item | Topic | Recommendation | Location (page / line) |
|---|---|---|---|
| **Title and abstract** | | | |
| 1 | Title and abstract | (a) Study design indicated in the title. (b) Balanced summary in the abstract. | Title (ln 2-3); Abstract (ln 10-29) |
| **Introduction** | | | |
| 2 | Background/rationale | Explain the scientific background and rationale for the investigation being reported. | Introduction, ln 34 |
| 3 | Objectives | State specific objectives, including any prespecified hypotheses. | Introduction, ln 61-62 |
| **Methods** | | | |
| 4 | Study design | Present key elements of study design early in the paper. | §2.1, ln 64 |
| 5 | Setting | Describe the setting, locations, and relevant dates, including periods of recruitment, exposure, follow-up, and data collection. | §2.1, ln 65–67 (single tertiary hospital; 2007–2022) |
| 6 | Participants | Give the eligibility criteria, and the sources and methods of selection of participants. | §2.1, ln 65-67; Figure 1 (flow), ln 76-77 |
| 7 | Variables | Clearly define all outcomes, exposures, predictors, potential confounders, and effect modifiers. | §2.3–2.5, ln 120-205 |
| 8 | Data sources / measurement | For each variable of interest, give sources of data and details of methods of assessment (measurement). | §2.2-2.4, ln 81-166 |
| 9 | Bias | Describe any efforts to address potential sources of bias. | §2.2, ln 111-114 |
| 10 | Study size | Explain how the study size was arrived at. | §2.5 ln 201 |
| 11 | Quantitative variables | Explain how quantitative variables were handled in the analyses; if applicable, describe which groupings were chosen and why. | §2.5, ln 173 |
| 12 | Statistical methods | (a) Describe all statistical methods, including those used to control for confounding. (b) Methods to examine subgroups and interactions. (c) How missing data were addressed. (d–e) Sensitivity analyses. | §2.5, ln 167-205 |
| **Results** | | | |
| 13 | Participants | Report numbers of individuals at each stage, reasons for non-participation, and consider a flow diagram. | Figure 1 (STROBE flow) |
| 14 | Descriptive data | Give characteristics of study participants and information on exposures and potential confounders. | §3.1, ln 207-215 |
| 15 | Outcome data | Report numbers of outcome events or summary measures. | §3.2, ln 219-225 |
| 16 | Main results | (a) Give unadjusted and confounder-adjusted estimates and their precision (e.g., 95% CI). (b) Report category boundaries when continuous variables were categorized. (c) Translate relative risk into absolute risk where meaningful. | §3.4, ln 240-276 |
| 17 | Other analyses | Report other analyses done—e.g., analyses of subgroups and interactions, and sensitivity analyses. | §3.4, ln 269-276 |
| **Discussion** | | | |
| 18 | Key results | Summarise key results with reference to study objectives. | Discussion, ln 282-287 |
| 19 | Limitations | Discuss limitations, taking into account sources of potential bias or imprecision. | Discussion (limitations), ln 350-379 |

| Item | Topic | Recommendation | Location (page / line) |
|---|---|---|---|
| 20 | Interpretation | Give a cautious overall interpretation of results considering objectives, limitations, multiplicity of analyses, results from similar studies, and other relevant evidence. | Conclusion, ln 380–388 |
| 21 | Generalisability | Discuss the generalisability (external validity) of the study results. | Discussion, ln 351-352 |
| **Other information** | | | |
| 22 | Funding | Give the source of funding and the role of the funders for the present study. | Funding statement (back matter) |

**Note:** This study is observational; reporting followed the STROBE guideline for cross-sectional studies. An information and explanation document is available from the STROBE Statement website.

## 2. Supplementary Figure S1

**Figure S1.** Regression diagnostics for the PAS mCSA model. Residual-versus-fitted plots, normal Q–Q plots, and residual histograms are shown for the untransformed outcome (panels A–C) and the natural-log-transformed outcome (panels D–F). Log-transformation improved the normality of the residuals (Shapiro–Wilk W: 0.87 → 0.94; skewness: 1.9 → −1.0) and removed the heteroscedasticity present in the untransformed model (Breusch–Pagan P: 0.01 → 0.95), supporting the log scale as the primary specification.

Supplementary Figure S1. Regression diagnostics for the PAS mCSA model (untransformed vs natural-log outcome)

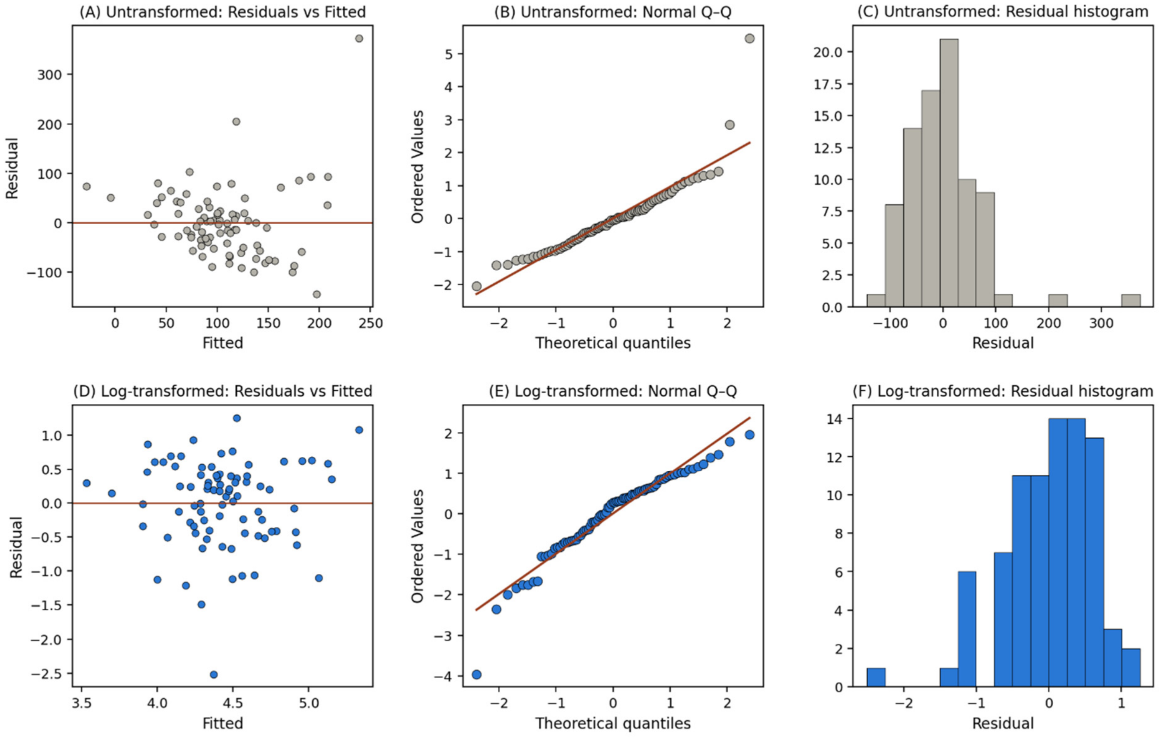

## 3. Supplementary Table S2

**Table S2.** Association between nasal cavity area and PAS mCSA across all combinations of exposure definition and outcome scale (multivariable linear regression, n = 83).

| **Exposure** | **Outcome scale** | **B (95% CI)** | **P** | **Standardized β** |
|---|---|---|---|---|
| Bilateral narrowest-section area | Log | 0.0035 (0.0008 to 0.0063) | 0.0120 | 0.26 |
| Bilateral narrowest-section area | Untransformed | 0.5235 (0.2168 to 0.8301) | 0.0011 | 0.33 |
| Single-side smallest mCSA | Log | 0.0068 (0.0003 to 0.0134) | 0.0413 | 0.22 |
| Single-side smallest mCSA | Untransformed | 1.1219 (0.3875 to 1.8563) | 0.0032 | 0.30 |

All models were adjusted for age, sex, and BMI. B, unstandardized coefficient per $mm^2$. The bilateral narrowest-section area on the log scale is the primary specification; the remaining rows are sensitivity analyses.

## 4. Supplementary Table S3

**Table S3.** Sensitivity of the nasal cavity–PAS association to adjustment for body size (multivariable linear regression, n = 83; outcome = log-transformed PAS mCSA).

| Additional adjustment | B (95% CI) | P | Std. β | Adj. $R^2$ | VIF |
|---|---|---|---|---|---|
| None (age+sex+BMI) | 0.0035 (0.0008–0.0063) | 0.012 | 0.26 | 0.168 | 1.02 |
| + Height | 0.0035 (0.0008–0.0063) | 0.012 | 0.26 | 0.173 | 1.02 |
| + Neck circumference | 0.0035 (0.0008–0.0063) | 0.013 | 0.26 | 0.157 | 1.02 |
| + Height<br>+ neck circumference | 0.0035 (0.0008–0.0063) | 0.013 | 0.26 | 0.162 | 1.02 |
| + Body surface area | 0.0035 (0.0008–0.0063) | 0.012 | 0.26 | 0.172 | 1.02 |

All models were adjusted for age, sex, and BMI. B, unstandardized coefficient per $mm^2$; Std. β, standardized coefficient for the nasal-area term; VIF, variance inflation factor for the nasal-area term; body surface area calculated by the Mosteller formula.

## 5. Supplementary Table S4

**Table S4.** Distribution of CT scans across scanner model and enrolment era (n=83)

| **Scanner** | **2007-2012** | **2013-2017** | **2018-2022** | **Total** |
|---|---|---|---|---|
| Philips Brilliance 64 | 2 | 4 | 37 | 43 |
| Philips iCT 256 | 3 | 4 | 17 | 24 |
| Philips Mx8000 IDT 16 | 6 | 4 | 0 | 10 |
| Philips Iqon Spectral CT | 0 | 0 | 3 | 3 |
| Philips Mx8000 | 2 | 0 | 0 | 2 |
| Siemens SOMATOM Definition Edge | 0 | 0 | 1 | 1 |
| **Total** | 13 | 12 | 58 | 83 |

Enrolment era is based on CT date. Eighty-two of 83 scans were acquired on Philips systems.

## 6. Supplementary Table S5

**Table S5.** Intra- and inter-observer reliability of the airway measurements (random subsample of 30 scans).

| Measurement | Intra ICC (95% CI) | Intra 95% LoA | Inter ICC (95% CI) | Inter 95% LoA |
|---|---|---|---|---|
| Bilateral narrowest nasal area ($mm^2$) | 0.999 | -3.7 to +3.4 | 0.999 | -4.4 to +5.0 |
| PAS mCSA ($mm^2$) | >0.999 | -3.6 to +4.0 | >0.999 | -3.1 to +4.1 |
| PAS lateral (mm) | 0.981 (0.96-0.99) | -2.5 to +2.8 | 0.974 (0.95-0.99) | -2.9 to +3.3 |
| PAS AP (mm) | 0.953 (0.90-0.98) | -1.9 to +2.0 | 0.953 (0.90-0.98) | -2.2 to +1.9 |

ICC, intraclass correlation coefficient (two-way random effects, absolute agreement, single measurement); LoA, Bland–Altman 95% limits of agreement. Mean bias was small throughout ($\leq$0.5 $mm^2$ for areas; $\leq$0.4 mm for linear measures).
